# Comparative volatilomics identifies ubiquitous sulfur compounds inhibiting the fungal pathogen *Rasamsonia argillacea*

**DOI:** 10.1128/spectrum.02666-25

**Published:** 2025-11-28

**Authors:** Delphine Adam, Djulia Bensaada, Nudzejma Stulanovic, Jean-François Focant, Pierre-Hugues Stefanuto, Sébastien Rigali

**Affiliations:** 1 InBioS—Center for Protein Engineering, Institut de Chimie B6a, University of Liège 220717, Liège, Belgium; 2 Molecular System – Organic Biological Analytical Chemistry Group, University of Liège 26658, Liège, Belgium; University of Melbourne, Melbourne, Australia

**Keywords:** volatilomics, antifungal compounds, cystic fibrosis, chronic granulomatous disease, sulfur-containing volatile compounds, *Aspergillus fumigatus*, *Candida albicans*

## Abstract

Members of the phylum Actinomycetota, particularly *Streptomyces* species, are prolific producers of bioactive metabolites, and bioprospecting in unique environments may uncover novel species producing previously undescribed antifungal compounds. In previous work, *Streptomyces* strains isolated from cave moonmilk deposits completely inhibited the growth of *Rasamsonia argillacea*, an emerging fungal pathogen associated with chronic granulomatous disease (CGD) and cystic fibrosis (CF). Cross-streak and bipartite Petri dish assays revealed that *R. argillacea* inhibition occurred specifically when bacteria were cultivated on Mueller-Hinton agar (MHA) and was mediated by volatile compounds rather than diffusible metabolites. This antifungal effect was not strain specific, as it was reproduced by phylogenetically diverse bacteria grown on MHA, suggesting the involvement of ubiquitous volatile molecules. Similar inhibitory effects were observed on MHA against other fungi and yeasts relevant to CGD and CF, supporting the hypothesis that these volatiles are broadly toxic rather than species-specific. To identify candidate ubiquitous antifungal VCs, we conducted comparative volatilomics of two phylogenetically distant bacterial strains that consistently inhibited fungal growth. Among the 143 VCs detected, only dimethyl trisulfide (DMTS) and dimethyl disulfide (DMDS) showed consistent presence in both bacterial volatilomes, with significantly increased production under conditions that promoted fungal inhibition. Exposure assays with pure compounds confirmed that both DMDS and DMTS strongly inhibited *R. argillacea* growth, with DMTS exhibiting greater potency. Our findings position these ubiquitous sulfur compounds as valuable models for exploring novel agents against human pathogens.

**IMPORTANCE:**

Our findings suggest that antifungal activity against human pathogens may arise from common metabolic pathways shared across diverse microbes rather than from unique biosynthetic systems. Because dimethyl disulfide and dimethyl trisulfide can also be generated through microbial and dietary sulfur metabolism, it is plausible that the human microbiome may produce similar volatiles depending on diet composition, particularly following consumption of sulfur-rich foods. This study, therefore, underscores the critical influence of culture conditions on revealing bioactive volatile production and opens intriguing perspectives on the ecological and physiological roles of ubiquitous microbial metabolites in regulating fungal colonization and microbiome-host interactions.

## INTRODUCTION

Given the rising threat of antibiotic resistance and the limited availability of effective treatments, bioprospecting for natural antifungals still represents a valuable strategy to find new leads to combat fungal infections (1–4). Exploring natural compounds from microbes, plants, and marine organisms offers the potential to identify novel antifungal agents that can inhibit fungal colonization, enhance immune responses, or disrupt biofilms that contribute to persistent infections (5–7). Among microbial sources, *Actinomycetota*, particularly *Streptomyces* species, have been one of the most successful producers of antimicrobial compounds (8, 9). Targeting extreme or highly specific environmental niches for the isolation of new representative members of these bacterial taxa is expected to yield new antifungal molecules (10, 11).

Two representative examples of such diseases where chronic and sometimes invasive fungal infections remain a major threat to individuals are chronic granulomatous disease (CGD) and cystic fibrosis (CF) (12). Several fungi and yeasts are recurrently associated with these diseases, including *Aspergillus spp*., *Scedosporium spp*., and *Candida spp*., with *Aspergillus fumigatus* being the most prevalent in both CF and CGD (13–15). Recently, *Rasamsonia* species, particularly *R. argillacea*, have emerged as important fungal pathogens in these patients, although their clinical manifestations differ between CGD and CF (16–18). In CGD, *Rasamsonia* has been increasingly reported as a cause of invasive fungal infections, exhibiting intrinsic resistance or reduced susceptibility to azole antifungals and posing significant treatment challenges. Amphotericin B and newer agents such as isavuconazole are sometimes effective, but therapeutic responses remain inconsistent (16, 19–21). In CF, *Rasamsonia* generally behaves as a chronic airway colonizer rather than an invasive pathogen (21, 22). The increasing recognition of *Rasamsonia* in both CGD and CF underscores the need for improved diagnostics, heightened clinical awareness, and novel antifungal strategies. Specifically, identifying natural compounds that can prevent fungal colonization, degrade CF mucus, or enhance immune defenses in CGD could lead to new and more effective treatment options.

In a previous investigation of the antifungal properties against pathogens commonly encountered in both CF and CGD, we observed that nearly all *Streptomyces* strains isolated from moonmilk deposits exhibited complete inhibition of *R. argillacea* growth (23, 24). This unexpected finding raised several critical questions: Is this inhibitory activity unique to strains from this environment, or is it a widespread bacterial trait? Which culture conditions most effectively induce this antifungal activity? Does the medium-dependent production of antifungal compounds specifically target *R. argillacea*? Is this inhibition mediated by volatile compounds (VCs), and if so, which molecules are responsible? To address these questions, we conducted a comprehensive investigation into the nature of this antifungal activity, aiming to uncover antifungal compounds that could possibly serve as effective agents for CF- and CGD-associated fungal infections.

## RESULTS AND DISCUSSION

### Assessment of media and strain dependence of antifungal production

**Fig 1:**
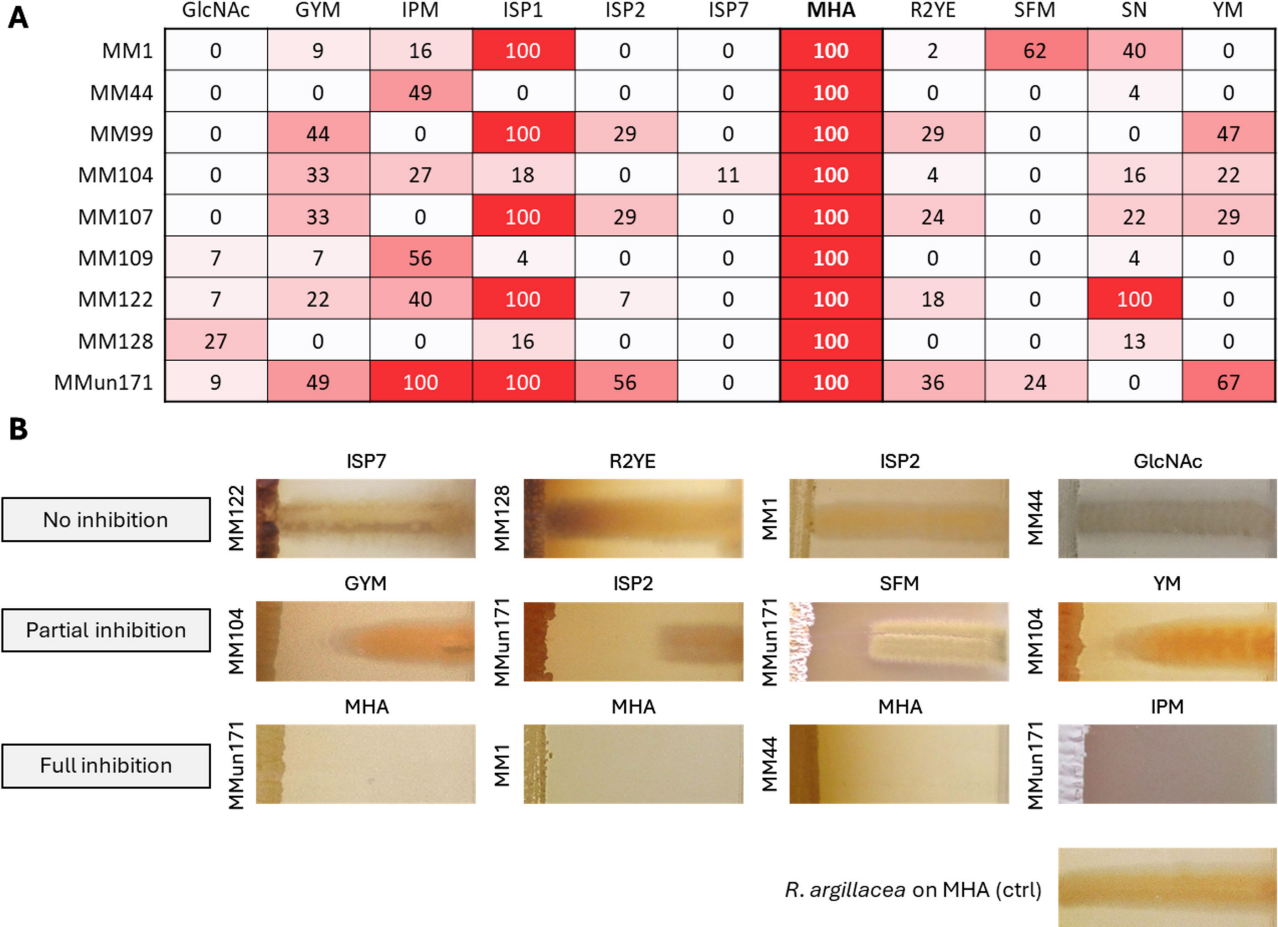
Medium-dependent production of antifungal compound(s) inhibiting *R. argillacea* growth. (**A**) Heatmap showing antifungal activity of actinomycetes against *R. argillacea* across different media. Numbers indicate the percentage (in %) of fungal growth inhibition, compared to the growth of the negative control. (**B**) Selected representative images of cross-streak assays showing no, partial, and full inhibition of *R. argillacea*.

As previously reported by Maciejewska et al., our collection of moonmilk-isolated *Streptomyces* strains exhibited strong growth inhibitory activity against *R. argillacea* when cultivated on Mueller-Hinton Agar (MHA) (24). To assess whether this inhibition was medium dependent, we tested nine strains of our strain collections (eight *Streptomyces* and one *Amycolatopsis* species [23, 24]), in cross-streak assays across 11 different media to identify the conditions supporting full inhibition (Fig. 1). As shown in Fig. 1, all tested strains produced compound(s) that caused growth inhibition of *R. argillacea*, with substantial variation in the observed antagonistic effects, depending on the strains and the culture conditions. We confirmed previous observations (24), as all tested strains completely inhibited the growth of *R. argillacea* on MHA (Fig. 1A). Notably, five of the nine strains (MM1, MM99, MM107, MM122, and MMun171) also caused significant growth inhibition on ISP1 medium, while the remaining four strains were harmless to the fungus under these conditions. Two strains, *Streptomyces* sp. MM122 and *Amycolatopsis* sp. MMun171, also exhibited strong antifungal activity on Starch Nitrate (SN) and Instant Potato Mash (IPM) media, respectively (Fig. 1). In contrast, all strains showed little to no antifungal activity on ISP7 medium.

**Fig 2:**
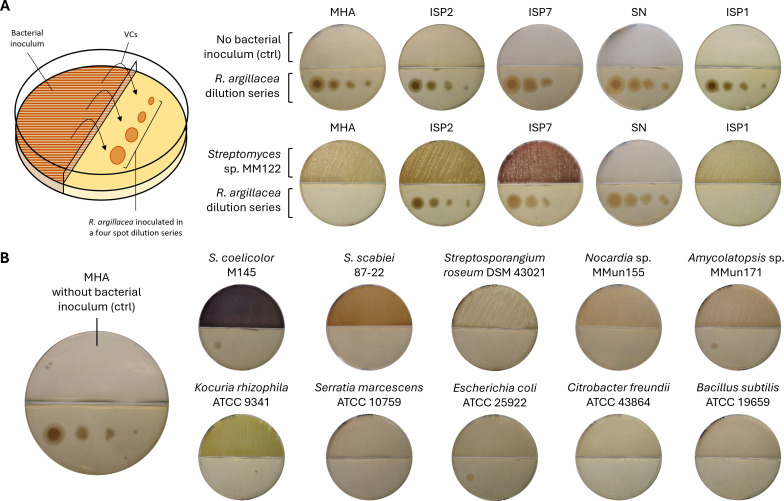
Evidence of MHA-mediated production of volatile antifungal(s) active against *R*. *argillacea*. (**A**) Bi-compartment Petri dish assay showing volatile-mediated inhibition by *Streptomyces* sp. MM122. The test strain was inoculated in the top compartment, while *R. argillacea* was inoculated in the other compartment as a four-spot dilution series (10², 10³, 10⁴, and 10⁶ CFU/mL) on Yeast Mold (YM) agar. Controls consisted of media without bacterial inoculation (top row). (**B**) Same experimental setup as in panel A, but restricted to MHA medium and using test bacteria from diverse genera, families, classes, and orders to assess broader phylogenetic contributions to volatile antifungal activity.

The complete inhibition of *R. argillacea* on MHA prompted us to investigate whether the lethal effect was mediated by VCs. A volatile fungicide would indeed saturate the space between the agar and plate lid, explaining the full inhibition (Fig. 1B, bottom panels). In contrast, a diffusible metabolite would display a gradient of inhibition, weakening with increased distance from the inoculation site (Fig. 1B, middle panels). To test this hypothesis, we conducted experiments using bipartite Petri dishes with a central barrier that blocked diffusion through agar but allowed volatiles to pass. When strain *Streptomyces* sp. MM122 was cultivated on MHA in one compartment and *R. argillacea* was inoculated as a dilution series (10², 10³, 10⁴, and 10⁶ CFU/mL) in the other, full fungal growth inhibition was observed (Fig. 2A). Full growth inhibition was also observed on ISP1 as anticipated from cross-streak assays. However, no inhibition occurred when *Streptomyces* sp. MM122 was inoculated on ISP2, ISP7, or SN media in the bipartite setup (Fig. 2A). The fact that no inhibition was observed when MM122 was grown in a bi-partite Petri dish containing SN demonstrates that this strain produces at least two different compounds capable of totally inhibiting the growth of the fungus. Indeed, as observed in Fig. 1A, MM122 grown on SN can totally inhibit the growth of *R. argillacea* when both microorganisms are inoculated in a single-compartment Petri dish, suggesting the existence of a diffusible potent antifungal compound. Interestingly, *Streptomyces* sp. MM122 was able to completely inhibit the growth of *R. argillacea* when cultured on ISP1 medium (Fig. 2A). The nutrient compositions of MHA and ISP1 are notably similar, both containing casein hydrolysates (17.5 g/L and 5 g/L, respectively) and either beef extract (3 g/L) or yeast extract (3 g/L), respectively. Additionally, MHA includes starch (1.5 g/L) as an extra carbon source. These similarities in nutrient composition likely explain the comparable bioactivity observed, with variations in activity strength potentially influenced by the quantity (e.g., casein hydrolysates) or source (e.g., tissue/cell extracts) of the nutrients or the presence of starch only in MHA.

Remarkably, similar results were observed on MHA for nearly all other tested strains in our collection, including *Streptomyces* species not isolated from cave deposits (e.g., *Streptomyces coelicolor* and *Streptomyces scabiei*), species belonging to other actinobacterial genera (*Amycolatopsis*, *Kocuria*, *Nocardia*, and *Streptosporangium*), and even non-actinobacterial species (*Escherichia coli*, *Citrobacter freundii*, *Serratia marcescens*, and *Bacillus subtilis*; Fig. 2B). These findings demonstrate that the fungicidal volatiles are not specific to a particular strain but are likely ubiquitous byproducts of bacterial metabolism during growth on MHA.

**Fig 3:**
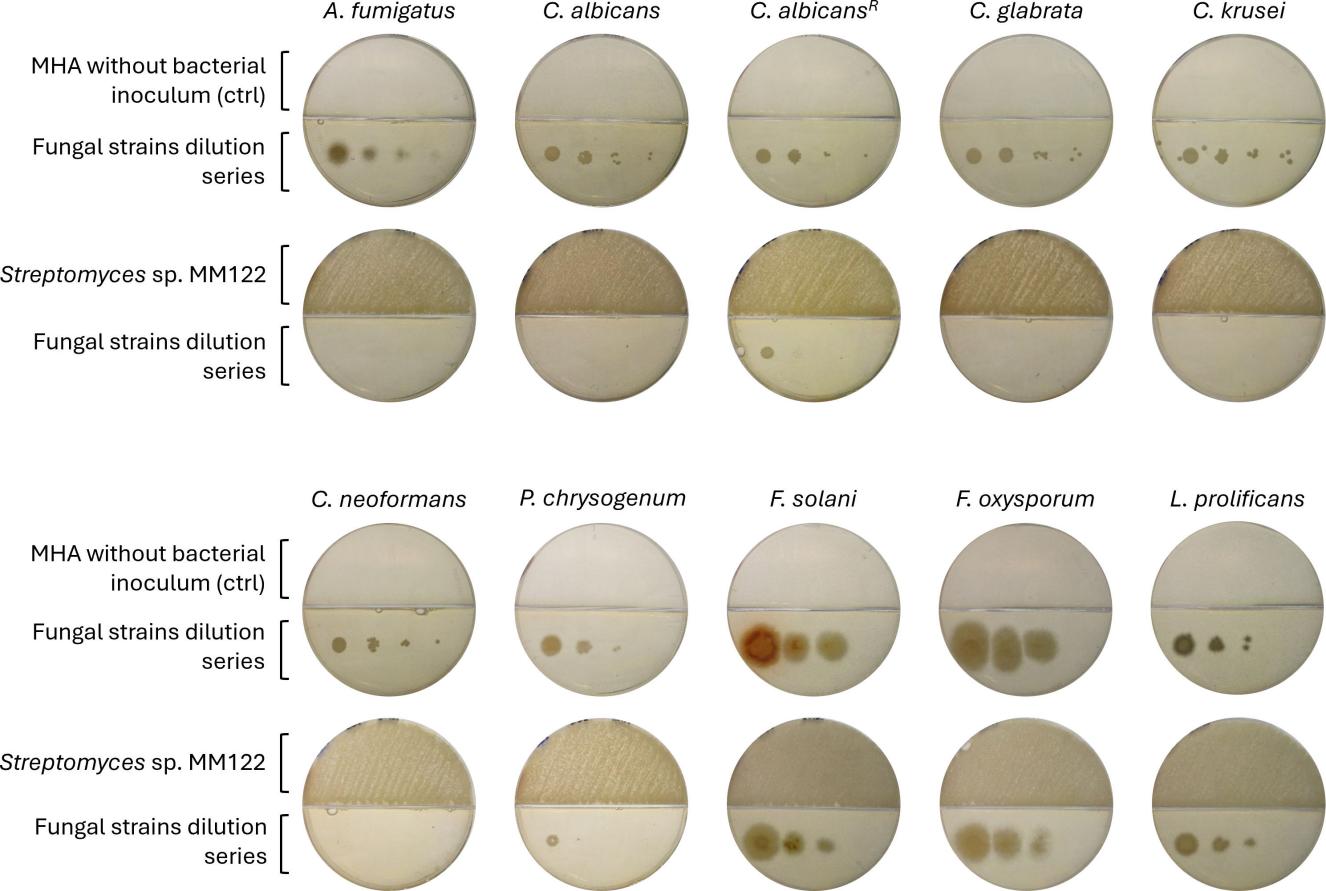
Assessment of fungi and yeasts sensitive to MHA-induced antifungal compounds. Evidence of MHA-induced production of volatile antifungal(s) active against a series of fungal and yeast species. The test strain *Streptomyces* sp. MM122 is inoculated in the top compartment of the bi-partite Petri dish, while test strains are inoculated in a four-spot dilution series (10^2^, 10^3^, 10^4^, and 10^6^ CFU/mL) in the other compartment (Yeast Mold [YM] agar medium). For sources, collection, and references of strains, refer to Table 1 in the Materials and Methods section. See the Materials and Methods section for details on tested fungi and yeast species.

Finally, the same tests using bipartite Petri dishes with *Streptomyces* sp. M122 cultured on MHA was performed against a series of other fungal and yeast species, including strains commonly associated with CGD and CF, such as *Aspergillus* spp., *Candida* spp., and *Lomentospora prolificans*. As shown in Fig. 3, complete growth inhibition was observed for *Aspergillus fumigatus*, *Candida albicans*, *Candida glabrata*, *Candida krusei*, and *Cryptococcus neoformans*. Partial growth inhibition was observed against an azole-resistant strain of *Candida albicans* and *Penicillium chrysogenum*. In contrast, limited inhibition was observed against *Fusarium solani*, *Fusarium oxysporum*, and *Lomentospora prolificans* (Fig. 3). These results suggest that the VCs produced by *Streptomyces* sp. M122 is not only a common bacterial metabolite but may also exert broad antifungal activity by targeting conserved cellular processes across phylogenetically diverse fungi and yeasts.

### Comparative volatilomics

The identification of the volatile fungicide(s) active against *R. argillacea* was carried out by headspace solid-phase microextraction two-dimensional gas chromatography coupled with time-of-flight mass spectrometry (HS-SPME GC×GC–TOFMS). Before conducting the comparative study, we confirmed that the VCs capable of inhibiting *R. argillacea* growth were also produced in Mueller-Hinton Broth (MHB, liquid MHA). This step was crucial to facilitate compound extraction and enable direct sample injection for GC×GC–TOFMS analysis (see Materials and Methods and Fig. S1 for a detailed description of the validation test).

The strain *Streptomyces* sp. MM122 was selected due to its high reproducibility in completely inhibiting the growth of *R. argillacea* when cultured on MHA (in MHB). The comparative analyses were conducted using samples of *Streptomyces* sp. MM122 grown in liquid ISP2 medium, where this strain did not produce VCs able to inhibit the growth of *R. argillacea*. The target VC(s) were therefore expected to meet the following criteria: (i) They must originate from the bacterial metabolic activity, displaying a significant increase in fold-change (FC) in the MHB samples inoculated with *Streptomyces* sp. MM122 compared to the non-inoculated MHB controls (FC ≥2 [Log2 FC ≥ 1], *P*-value ≤ 0.05). (ii) They must show higher production levels or be exclusively produced when *Streptomyces* sp. MM122 is grown in MHB compared to their levels when the strain is cultured in ISP2 medium (the variation of the normalized abundance (NA) in MHB compared to ISP2 must be >0). (iii) Since the growth inhibition of *R. argillacea* was observed for all strains tested when grown on MHA, it is likely that at least one of the toxic VCs is a ubiquitous molecule produced as a result of the bacterial metabolism under this culture condition.

**Fig 4:**
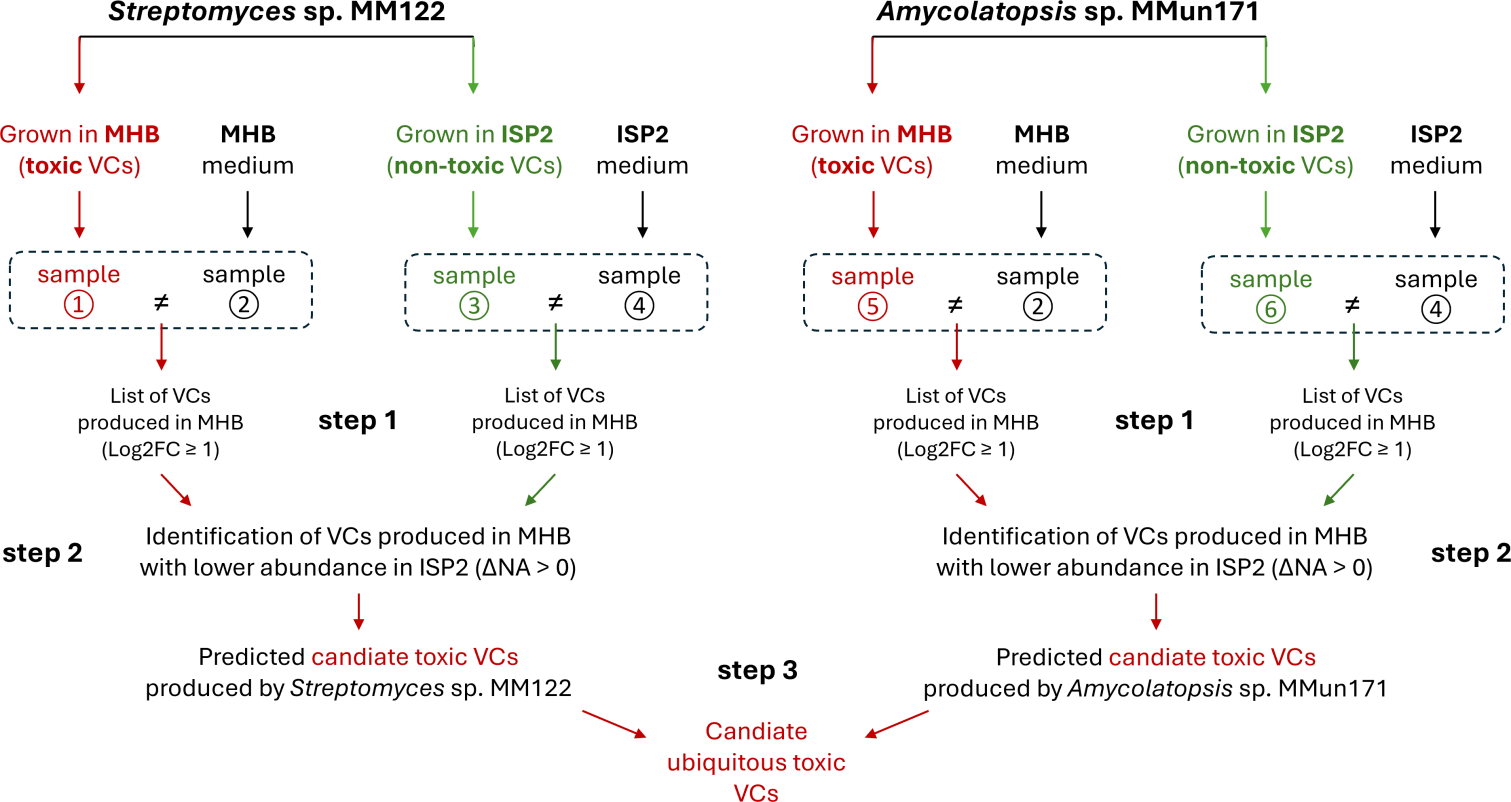
Experimental design for identifying candidate toxic VCs produced by *Streptomyces* sp. MM122 and *Amycolatopsis* sp. MMun171. Both organisms were cultured in two media: MHB (toxic VCs) and ISP2 (non-toxic VCs). Step 1: the pairwise comparison of the Log2 FC between samples ① and ②, and between samples ⑤ and ②, allows the identification of compounds produced in MHB by *Streptomyces* sp. MM122 and *Amycolatopsis* sp. MMun171, respectively. Step 2: the variation of the NA (∆NA) of compounds identified in step 1 allows the identification of compounds more abundant in MHB (medium with toxic VCs) compared to their levels in ISP2 (medium without VC-mediated toxicity). Step 3: identification of compounds common to both *Streptomyces* sp. MM122 and *Amycolatopsis* sp. MMun171.

To facilitate the identification of such compounds, the comparative GC×GC–TOFMS analysis was also performed using another strain, *Amycolatopsis* sp. MMun171 (23). Though both Actinomycetota, this strain is phylogenetically distant from *Streptomyces* sp. MM122, suggesting different metabolic responses and volatilome patterns, thereby facilitating the identification of ubiquitous bioactive compound(s) common to both strains. These compounds are expected to show increased production levels, specifically in MHB, for both bacterial strains. The experimental design for identifying candidate toxic VCs is illustrated in Fig. 4.

**Fig 5:**
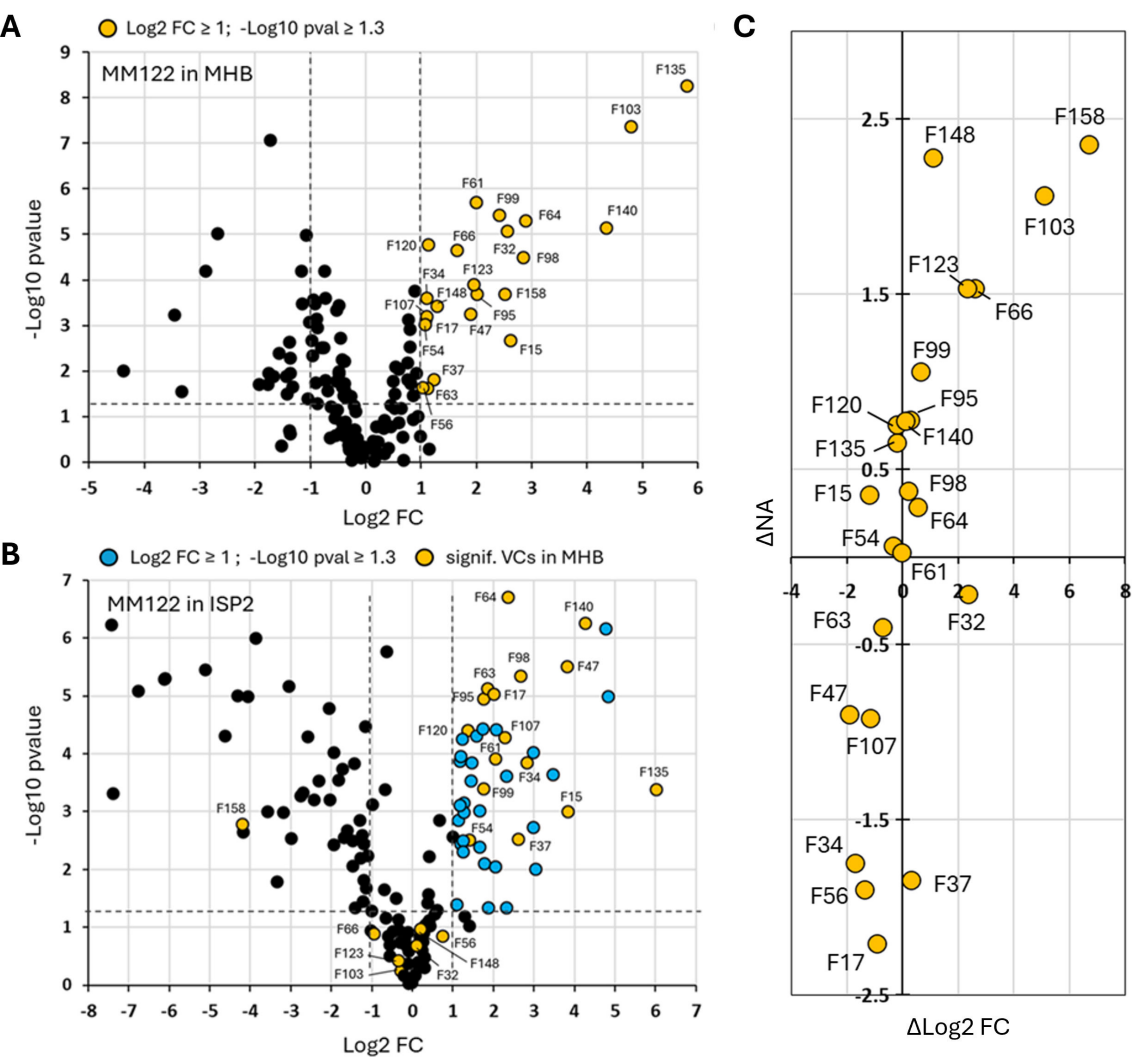
Identification of candidate toxic VCs produced by *Streptomyces* sp. MM122. (**A**) VCs resulting from the metabolic activity of *Streptomyces* sp. MM122 in the MHB medium. Significant VCs (yellow circles) exhibit a Log2 FC ≥ 1 and a −Log10 *P*-value ≥ 1.3 (*P* ≤ 0.05). (**B**) VCs resulting from the metabolic activity of *Streptomyces* sp. MM122 in the ISP2 medium. Significant VCs exhibit a Log2 FC ≥1 and a −Log10 *P*-value ≥ 1.3 (*P* ≤ 0.05). The VCs only significantly produced by *Streptomyces* sp. MM122 in the ISP2 medium are marked as blue circles, and VCs significantly produced in the MHB medium are marked as yellow circles. (**C**) Combined variation of the metabolic activity (∆log2FC) of VCs produced by *Streptomyces* sp. MM122, and the variation of the NA (∆NA) of each VC according to the toxic (MHB) vs the non-toxic (ISP2) cultivation media.

**Fig 6:**
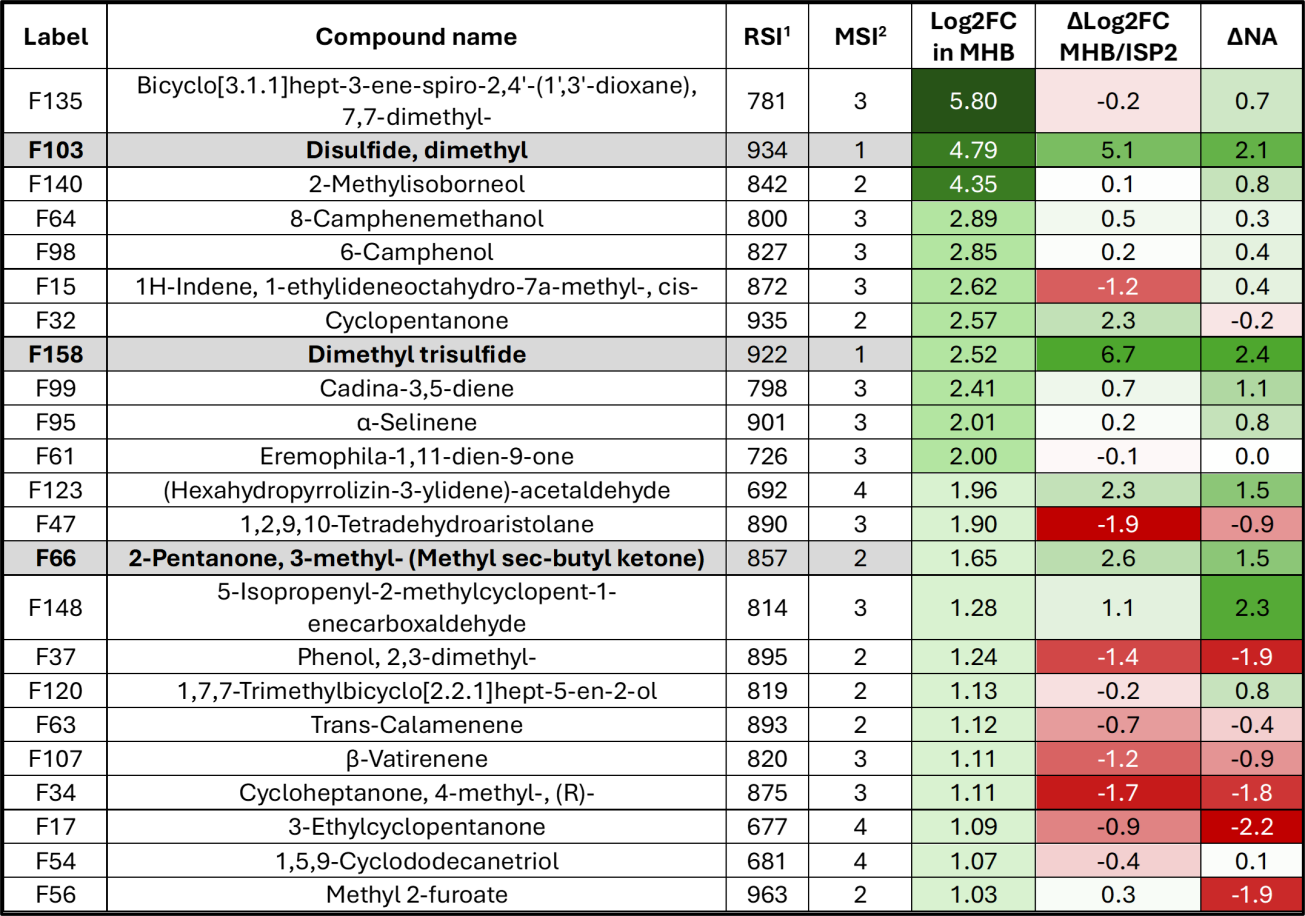
VCs resulting from the metabolic activity of *Streptomyces* sp. MM122 grown in MHB. Heatmap color code: red, reduced abundance (∆NA < 0) or reduced metabolic activity (∆Log2 FC < 0) in MHB compared to ISP2 medium; green, higher abundance (∆NA > 0) or increased metabolic activity (∆Log2 FC > 0) in MHB compared to ISP2 medium. ^1^RSI, Reverse Similarity Index; ^2^MSI: Identification levels based on the Metabolomics Standards Initiative (from level 1 [highest confidence] to level 4 [lowest], using criteria detailed in the Materials and Methods section). Compounds commonly found in extracts of both MM122 and MMun171 strains are highlighted in bold.

We first analyzed the compounds whose production was stimulated during the growth of *Streptomyces* sp. MM122 in MHB medium. Among the 143 identified VCs that passed the quality control (QC) filters, 23 exhibited a Log2 FC ≥ 1 compared to their levels in the non-inoculated medium, indicating that these molecules are likely products of the metabolic activity of *Streptomyces* sp. MM122 in the MHB medium (Fig. 5A and 6). In the next step, we examined the production levels of these 23 VCs in the ISP2 medium, under the assumption that the biosynthesis of toxic VCs would not be activated or at least less induced in conditions where *Streptomyces* sp. MM122 does not inhibit the growth of *R. argillacea*. Sixteen of these compounds also exhibited a Log2 FC ≥ 1 in the ISP2 medium, suggesting that they also likely result from the metabolic activity of *Streptomyces* sp. MM122 under this culture condition (Fig. 5B). Six compounds showed no significant modification of their production profiles, indicating that the nutrient composition of ISP2 does not seem to activate their biosynthesis. Remarkably, one compound (F158) displayed a marked decrease in fold change (Log2 FC = −4.18) compared to the non-inoculated ISP2 medium (Fig. 5B).

Finally, we compared the normalized abundance (NA) of the 23 VCs between the conditions that do inhibit (i.e., MHB medium) and do not inhibit (i.e., ISP2 medium) the growth of *R. argillacea*. The rationale is that the abundance of the candidate toxic VCs must be higher in the MHB medium, where we observed the inhibition of *R. argillacea*. Among these 23 VCs, eight compounds showed reduced abundance in MHB compared to ISP2 medium (∆NA < 0) and therefore are unlikely to be the candidate compounds responsible for the cytotoxic activity observed in MHB medium. Nine VCs displayed a moderate increase in abundance (0 < ∆NA < 1) between the two culture conditions, while six compounds demonstrated a significant increase in abundance (∆NA > 1) in MHB medium (Fig. 6).

A correlation between stimulated biosynthesis (∆Log2 FC > 0) and higher abundance (∆NA > 0) under conditions inhibiting the growth of *R. argillacea* was observed for 11 VCs (Fig. 6). The molecules positioned in the top-right quadrant of the scatter plot (Fig. 5C) represent the VCs that best satisfy the two criteria for the candidate toxic molecules produced by *Streptomyces* sp. MM122. This visual approach helped to identify two compounds that stood out distinctly based on their positioning on the scatter plot: F158 (dimethyl trisulfide, DMTS) and F103 (dimethyl disulfide [DMDS]). DMDS (F103) and DMTS (F158) are volatile sulfur-containing organic compounds, and both have been studied for their antifungal properties (25–32). Their mechanisms of action primarily involve the production of reactive sulfur species, which can interfere with fungal cell function and integrity. The other three VCs deserve further attention due to their position on the scatter plot: F66 (2-pentanone, 3-methyl-[methyl sec-butyl ketone]), F123 ([hexahydropyrrolizin-3-ylidene]-acetaldehyde), and F148 (5-isopropenyl-2-methylcyclopent-1-enecarboxaldehyde). Methyl sec-butyl ketone (F66) has been identified in the volatilome of *Bacillus* species with demonstrated inhibitory activity against *Fusarium solani* (33). Its antifungal properties against *Paecilomyces lilacinus* and *Clonostachys rosea* have been directly evaluated using the pure compound, showing inhibitory activity at high concentration (34). (Hexahydropyrrolizin-3-ylidene)-acetaldehyde (F123) has never been specifically tested, as antifungal agent, but such bioactivity has been reported for other bicyclic nitrogen-containing compounds (e.g., pyrrolizidine alkaloids) (35). Similarly, 5-isopropenyl-2-methylcyclopent-1-enecarboxaldehyde (F148) has not been investigated for its antifungal properties, though such bioactivity has been reported for structurally similar monoterpene aldehydes (36).

**Fig 7:**
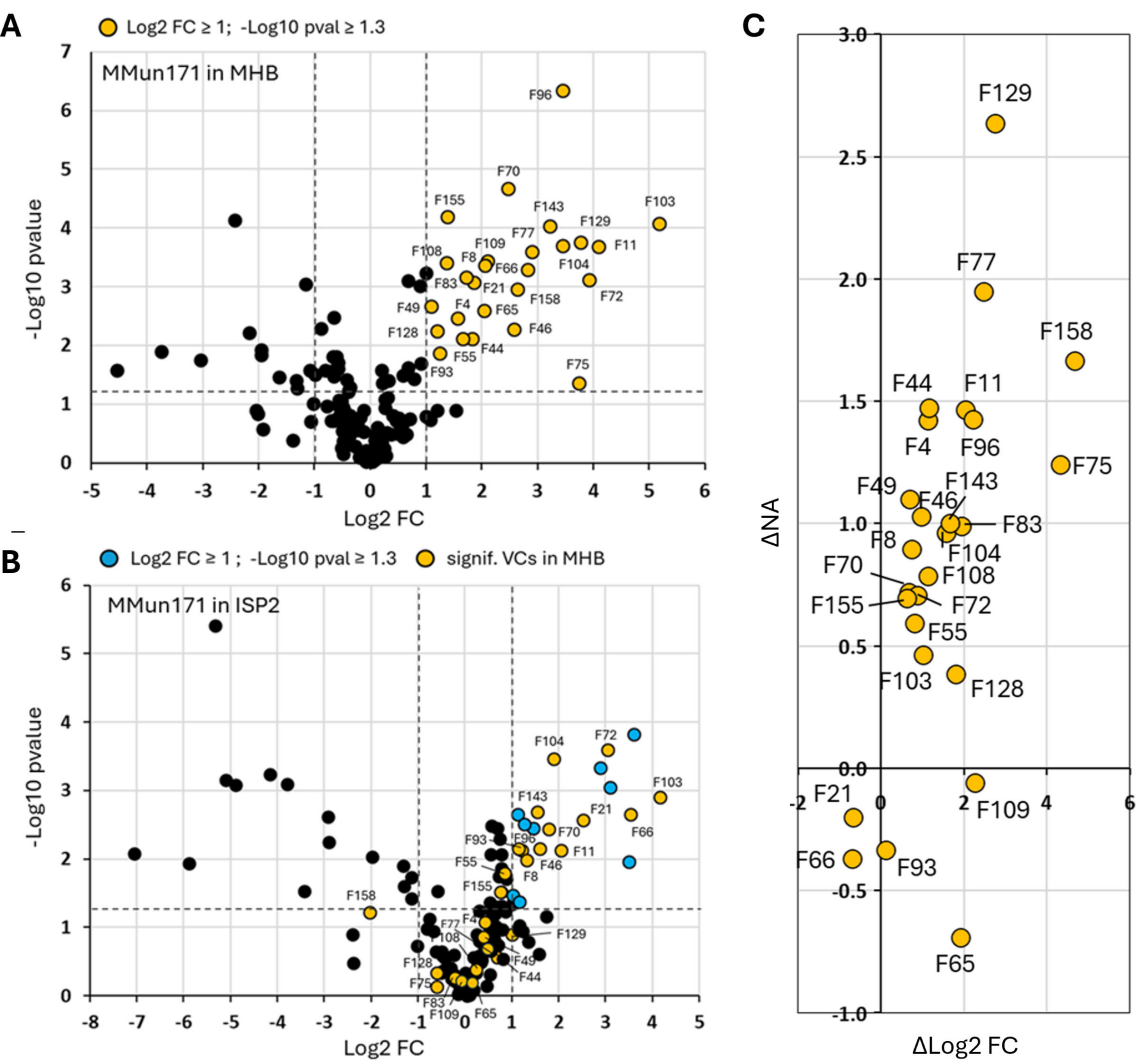
Identification of candidate toxic VCs produced by *Amycolatopsis* sp. MMun171. (**A**) VCs resulting from the metabolic activity of *Amycolatopsis* sp. MMun171 in the MHB medium. Significant VCs (yellow circles) exhibit a Log2 FC ≥ 1 and a −Log10 *P*-value ≥ 1.3 (*P* ≤ 0.05). (**B**) VCs resulting from the metabolic activity of *Amycolatopsis* sp. MMun171 in the ISP2 medium. Significant VCs exhibit a Log2 FC ≥ 1 and a −Log10 *P*-value ≥ 1.3 (*P* ≤ 0.05). The VCs only significantly produced by *Amycolatopsis* sp. MMun171 in the ISP2 medium are marked as blue circles, and VCs significantly produced in the MHB medium are marked as yellow circles. (**C**) Combined variation of the metabolic activity (∆log2FC) of VCs produced by *Amycolatopsis* sp. MMun171, and the variation of the normalized abundance (∆NA) of each VC according to the toxic (MHB) vs the non-toxic (ISP2) cultivation media.

**Fig 8:**
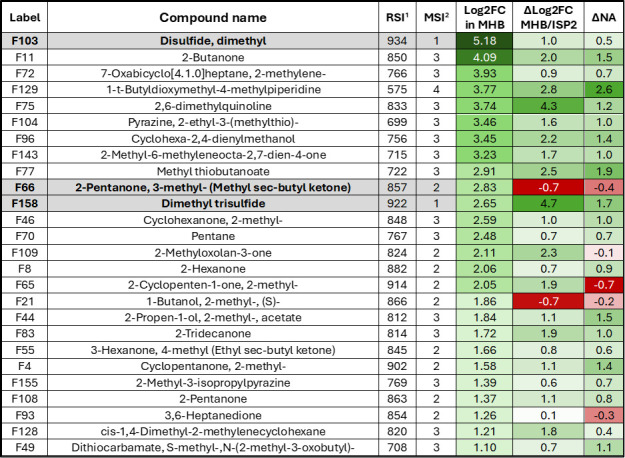
VCs resulting from the metabolic activity of *Amycolatopsis* sp. MMun171 grown in MHB. Heatmap color code: red, reduced abundance (∆NA < 0) or reduced metabolic activity (∆Log2 FC < 0) in MHB compared to ISP2 medium; green, higher abundance (∆NA > 0) or increased metabolic activity (∆Log2 FC > 0) in MHB compared to ISP2 medium. ^1^RSI, Reverse Similarity Index; ^2^MSI: Identification levels based on the Metabolomics Standards Initiative (from level 1 [highest confidence] to level 4 [lowest], using criteria detailed in the Materials and Methods section). Compounds commonly found in extracts of both MM122 and MMun171 strains are highlighted in bold.

The same experimental design was carried out with samples of *Amycolatopsis* sp. MMun171. Twenty-six VCs exhibited a Log2 FC ≥ 1 compared to their levels in the non-inoculated medium, indicating that these molecules are likely products of the metabolic activity of *Amycolatopsis* sp. MMun171 in the MHB medium (Fig. 7A and 8). In the ISP2 medium, 12 of these 26 compounds also exhibited a Log2 FC ≥ 1, while 13 compounds did not show significant modified production profiles (Fig. 7B). One compound (F158) displayed a marked decreased fold change (Log2 FC = −2.03) compared to the non-inoculated ISP2 medium, as earlier observed for *Streptomyces* sp. MM122 (Fig. 7B). The comparison of the normalized abundance of the 26 VCs highlighted 10 compounds with a significant increase in abundance (∆NA > 1) in MHB medium compared to the ISP2 medium (Fig. 7C).

Twenty-one compounds showed a correlation between stimulated biosynthesis (∆Log2 FC > 0) and higher abundance (∆NA > 0) under conditions inhibiting the growth of *R. argillacea* (Fig. 8). Four of these VCs best satisfy the two criteria for the candidate toxic molecules produced by *Amycolatopsis* sp. MMun171 according to their positioning on the scatter plot: F75 (2,6-dimethylquinoline), F77 (methyl thiobutanoate), F129 (1-t-butyldioxymethyl-4-methylpiperidine), and F158 (DMTS). While quinoline derivatives have a broad range of biological activities, including antifungal properties, 2,6-dimethylquinoline itself is not primarily recognized as a significant antifungal agent. Similarly, methyl thiobutanoate and 1-t-butyl-dioxymethyl-4-methylpiperidine are not currently classified as significant antifungal compounds. Instead, as stated above, DMTS can exhibit antifungal properties. Importantly, only three compounds were common in both *Streptomyces* sp. MM122 and *Amycolatopsis* sp. MMun171 volatilomes under conditions that inhibit the growth of *R. argillacea*: F66 (methyl sec-butyl ketone), F103 (DMDS), and F158 (DMTS). Of these, only DMTS and DMDS met both criteria—stimulated biosynthesis and higher abundance—in both tested bacteria and could therefore be the searched candidate ubiquitous VCs that would be responsible for the observed growth inhibition.

### Toxicity assessment of DMDS and DMTS on *Rasamsonia* species

**Fig 9:**
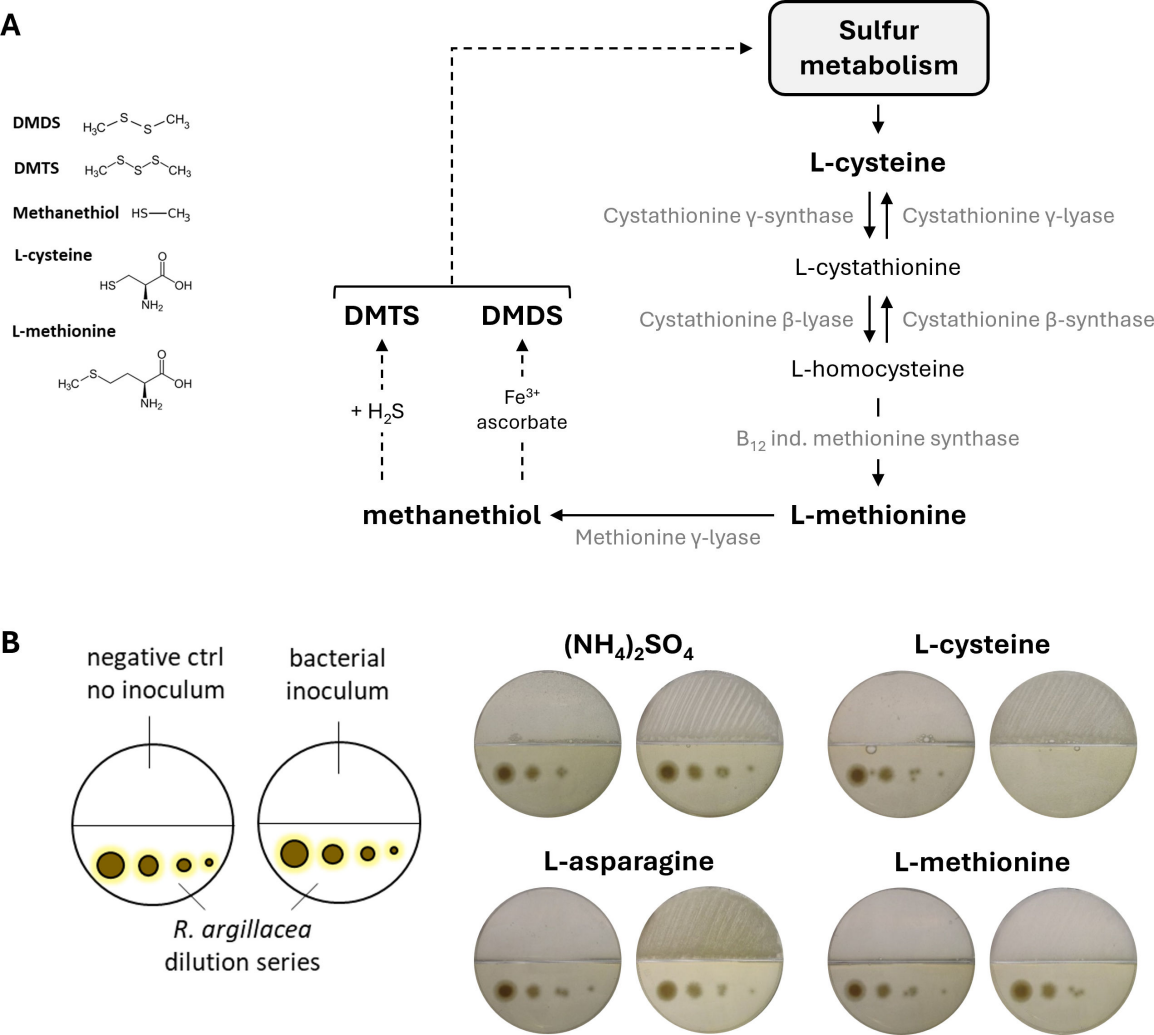
Predicted metabolic pathway for DMDS and DMDS synthesis and effect of sulfur-containing amino acids catabolism on the growth of *R*. *argillacea*. (**A**) Catabolism of sulfur-containing amino acids and metabolic pathway to generate DMDS and DMTS. (**B**) Antifungal VCs production assays with strain MM122 grown on minimal medium with different nitrogen sources.

DMDS and DMTS are volatile organic compounds derived from the catabolism of sulfur-containing amino acids, L-cysteine and L-methionine, through the oxidation of methanethiol (Fig. 9A) (37–39). Analysis of the amino acid composition of MHA medium (beef extract and casein hydrolysate) confirmed the presence of cystine (oxidized cysteine dimer) and methionine, potential sources of DMDS and DMTS (BD Bionutrients Technical Manual). Additionally, methanethiol, reported as a VC from MHA (40), provides another precursor for bacterial production of these compounds.

To test whether toxic sulfur-containing VCs in MHA were generated via amino acid catabolism, antifungal assays were repeated using bipartite Petri dishes with a minimal medium (MM) supplemented with either L-cysteine or L-methionine as the sole nitrogen source (Fig. 9B). When cultivated on MM supplemented with L-cysteine, *Streptomyces* sp. MM122 and *Amycolatopsis* sp. MMun171 fully inhibited *R. argillacea* growth, confirming the role of cysteine catabolism in the production of sulfur-containing VCs (Fig. 9B). Supplementation with L-methionine resulted in weak inhibition of *R. argillacea* growth, limited to the lowest fungal dilution (Fig. 9B). The weaker inhibition is likely due to sub-lethal sulfur-containing VCs concentrations caused by a lower bacterial capacity to import and catabolize methionine compared to cysteine, as deduced from the fact that our strains grew much less well than on medium with L-cysteine. In contrast, no inhibition was observed with inorganic nitrogen ([NH_4_]_2_SO_4_) or L-asparagine, which do not generate bioactive sulfur-containing VCs (Fig. 9B).

**Fig 10:**
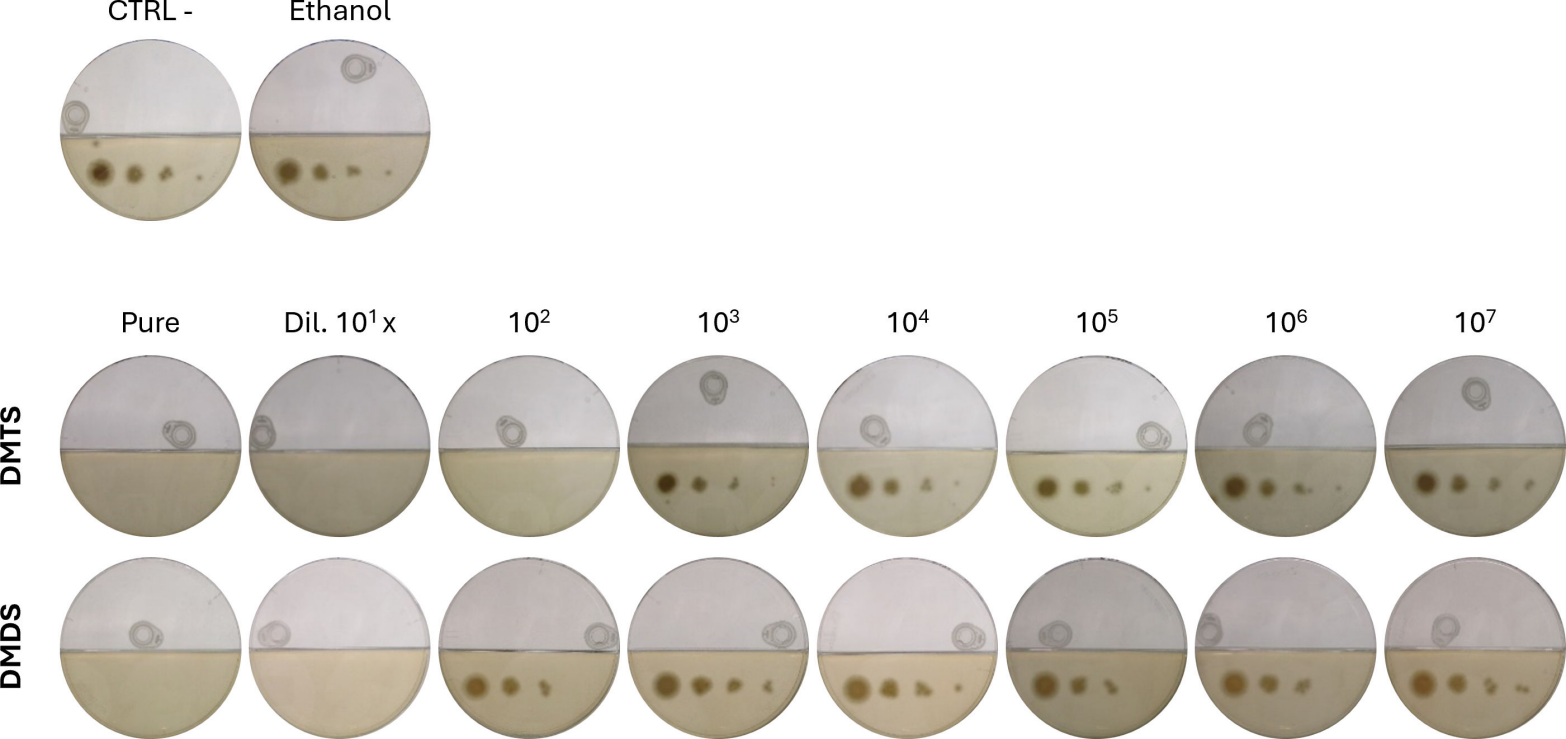
Assessment of *R*. *argillacea*’s susceptibility to DMTS and DMDS. A total of 100 µL of different dilutions of DMDS or DMTS is placed in a cap of a 2 mL tube in the empty top compartment of the bi-partite Petri dishes, while *R. argillacea* is inoculated in a four-spot dilution series (10^2^, 10^3^, 10^4^, and 10^6^ CFU/mL) in the other compartment (on Yeast Mold [YM] medium). An empty cap and a cap with ethanol (the solvent used for dilutions) were used as controls (top line).

To determine the specific toxicity of DMDS and DMTS, *R. argillacea* was exposed to eight gradual concentrations of each sulfur-containing VC using bipartite Petri dishes (Fig. 10). To prevent cross-contamination, Parafilm-sealed dishes containing either DMDS or DMTS were placed in separate incubators, and each dish was placed in its own individual airtight container to completely eliminate the risk of cross-contamination. Both DMTS and DMDS exhibited full growth inhibition of *R. argillacea*, with DMTS displaying inhibiting activity at concentrations ranging from 10.0−100 nmol/cm^3^ (Fig. 10). DMDS showed more moderate toxicity, fully inhibiting *R. argillacea* growth only at the two highest concentrations (11.8 and 1.18 µmol/cm^3^). These values are in line with the MIC of DMTS and DMTS tested against wheat fungal pathogen with lower MIC values of 58.7 nmol/cm^3^ for DMTS vs 352.5 nmol/cm^3^ for DMDS (25). Importantly, the reported concentrations represent the theoretical maximum average values, assuming complete volatilization of DMTS and DMDS into the headspace. In practice, the actual gas-phase concentration will be significantly lower due to equilibrium dynamics between the liquid phase, headspace vapor, and adsorption to surrounding surfaces, including retention within the agar.

**Fig 11:**
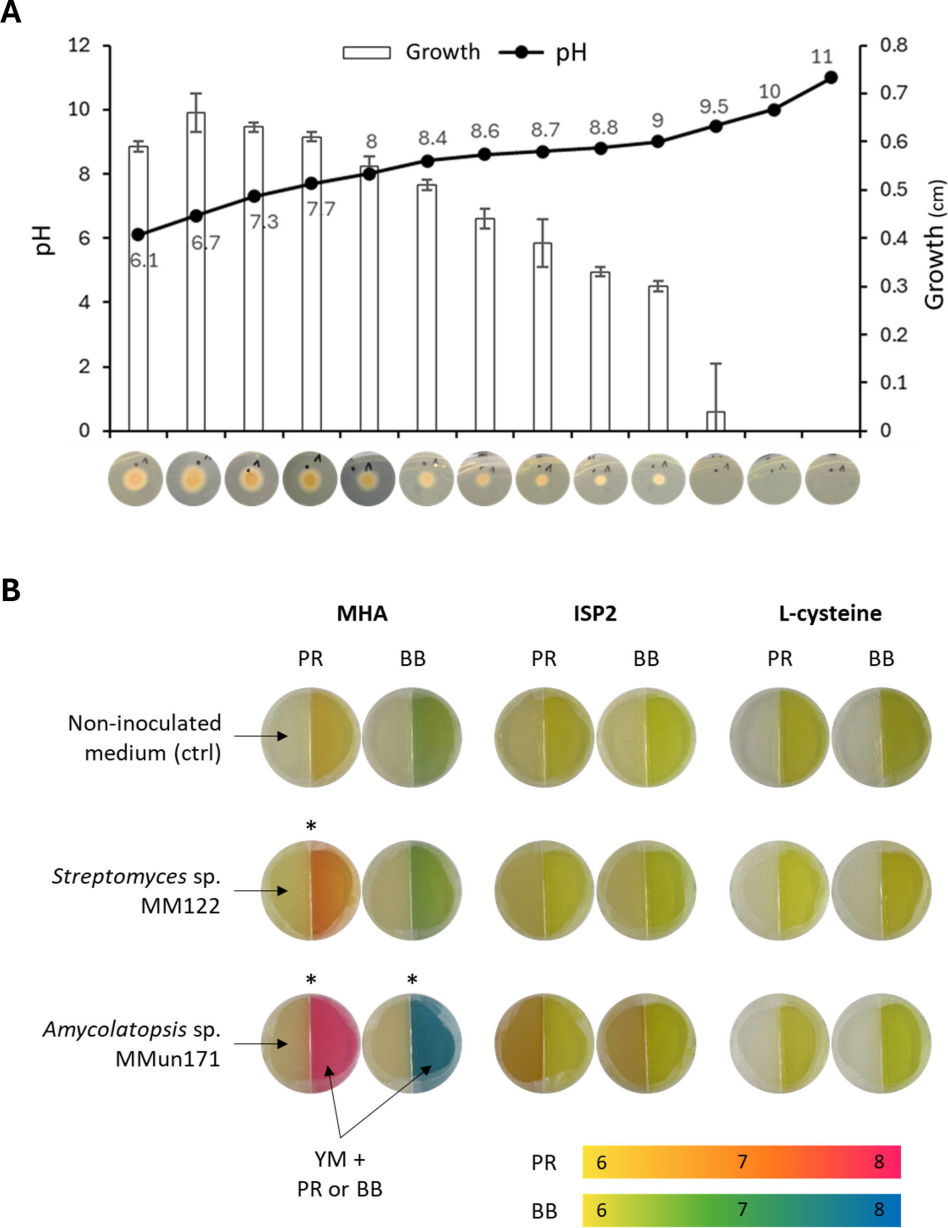
pH effect on the growth of *R*. *argillacea*. (**A**) Growth on the YM medium is measured as the average spot size (cm) of the *R. argillacea* inoculum. (**B**) Medium alkalinization as potential evidence of NH_3_ production *Streptomyces* sp. MM122 and *Amycolatopsis* sp. MMun17. pH indicators phenol red (PR) and bromothymol blue (BB) were used in the compartment separated from the bacterial culture. * Indicates conditions where alkalinization of the medium was observed.

Another compound potentially contributing to the observed inhibition is ammonia (NH_3_), which is a strong candidate due to its co-production with DMTS and DMDS during the degradation of peptides containing sulfur from methionine and cysteine. NH_3_ can inhibit microbial growth by increasing the pH of its surroundings, creating an alkaline environment that can be detrimental to microorganisms sensitive to high pH levels (41). When cultivated on Yeast Mold (YM) medium, *R. argillacea* exhibited tolerance to a pH range of 6.0−9.0, with noticeable growth impairment starting around pH 8.0 (Fig. 11A).

To assess medium alkalinization as potential evidence of NH_3_ production, the pH indicators phenol red and bromothymol blue were used in the compartment separated from *Streptomyces* sp. MM122 and *Amycolatopsis* sp. MMun171 cultures. Both strains were grown under conditions that either promoted (MHA or MM + L-cysteine) or did not promote (ISP2) the inhibition of *R. argillacea*. A slight alkalinization was observed only on MHA, where pH levels reached approximately 7.5−8.0 (Fig. 11B). This suggests that under these culture conditions, medium alkalinization could contribute—alongside VCs—to the inhibition of *R. argillacea*. Surprisingly, the amount of NH₃ produced in MM + L-cysteine was insufficient to modify the pH (Fig. 11B), indicating that under these conditions, alkalinization does not play a role in the observed growth inhibition.

### Conclusions

The motivation for this study stemmed from the striking observation that almost all *Streptomyces* strains isolated from moonmilk deposits completely inhibited the growth of *R. argillacea*, an emerging fungal pathogen associated with CGD and CF. This finding prompted key questions regarding the specificity of the microbial interactions involved, the influence of culture conditions, and the nature of the inhibitory compounds. Our study provides important insights into these aspects. We identified MHA as the culture medium most effective in triggering complete inhibition of *R. argillacea* growth. Importantly, we showed that this inhibition is mediated by volatile compounds, and this phenomenon was observed with any tested bacterial strain from diverse genera, families, classes, and orders when grown on MHA. Using HS-SPME GC×GC–TOFMS, we identified DMTS and DMDS as the most likely antifungal VCs responsible for this inhibitory effect. Assays with each pure molecule confirmed their ability to inhibit *R. argillacea* growth, with DMTS exhibiting a stronger effect than DMDS. Researchers conducting cross-streak or volatile-mediated inhibition assays on this medium should be aware that the observed antifungal effects are very likely, at least in part, mediated by these volatile sulfur-containing compounds.

Previous studies, mainly across plant pathogens, have reported that DMDS and especially DMTS exhibit broad-spectrum antifungal effects (25–28, 42, 43). It has also been shown that specific members of the *Burkholderiaceae* family contribute to soil suppressiveness against plant pathogens through the production of antifungal sulfurous volatile compounds linked to genes encoding dimethyl sulfoxide reductase and cysteine desulfurase (44). DMDS is a fumigant registered in parts of the U.S. for its high pesticidal activity (45, 46). Much fewer investigations have been performed with sulfur-containing compounds against human pathogens (43, 47). Both DMDS and DMTS act mainly by disrupting fungal cell membranes, leading to leakage and cell death. DMDS also inhibits ergosterol synthesis and respiration, while DMTS induces oxidative stress and organelle damage (26–28). While our findings underscore the potent and broad-spectrum antifungal activity of DMDS and DMTS, the therapeutic potential of these volatiles remains constrained. Several toxicological studies have shown that DMDS and related compounds can cause severe respiratory effects in animal models (48, 49), precluding their application in inhalation-based therapies.

## MATERIALS AND METHODS

### Strains and culture conditions

The microbial strains used in this study are listed in Table 1. *Streptomyces* and other actinobacteria were preserved as glycerol mycelium stocks at −80°C and −20°C. Bacterial and fungal strains were also preserved as glycerol stocks at −80°C and −20°C. The cultivation media used were (for 1 L): International *Streptomyces* Project (ISP) media (No. 1, 2, and 7) prepared according to Shirling and Gottlieb (50); YM medium (Yeast extract (VWR) 3 g, Bacto Malt Extract (Gibco) 3 g, D(+)-glucose monohydrate (Carl Roth) 10 g, Bacteriological Peptone (Condalab) 5 g, and Bacto Agar (BD Difco) 20 g; R2YE, MM, and SFM (Soy Flour Mannitol) media were prepared according to the protocol described in (51); MM was supplied with 25 mM N-acetylglucosamine as carbon source; IPM medium consists of 50 g of Maggi Mousline (Nestlé) powder and 12 g of Bacto Agar (BD Difco) dissolved in tap water; TSA (Tryptic Soy Broth (MerckMilipore) 30 g and Bacto Agar (BD Difco) 20 g; MHA (Mueller-Hinton Broth II (MerckMilipore) 22 g and Bacto Agar (BD Difco) 20 g; SN medium was prepared according to the formulation described in reference 52. Glucose, yeast, and malt (GYM) *Streptomyces* medium consists of glucose (4 g), yeast extract (4 g), malt extract (10 g), CaCO_3_ (2 g), and pH adjusted to 7.2 before adding 20 g of Bacto Agar (BD Difco). MM supplemented with different nitrogen sources (5 mM of L-methionine, L-cysteine, L-asparagine, or ammonium sulfate) was used for production assays of sulfur-containing VCs.

**TABLE 1:** Strains used in this study

Name (genus, species, strain)	Culture collection no	Experiment	Source^*a*^
Bacterial strains			
*Amycolatopsis* sp. MMun171	–^*b*^	Cross-streak assays, VC assays	CIP, (23)
*Nocardia* sp. MMun155	–	VC assays	CIP, (23)
*Streptomyces* sp. MM1	–	Cross-streak assays	CIP, (24)
*Streptomyces* sp. MM44	–	Cross-streak assays	CIP, (24)
*Streptomyces* sp. MM99	–	Cross-streak assays	CIP, (24)
*Streptomyces* sp. MM104	–	Cross-streak assays	CIP, (24)
*Streptomyces* sp. MM107	–	Cross-streak assays	CIP, (24)
*Streptomyces lunaelactis* MM109^T^	DSM 42149	Cross-streak assays	CIP, (53)
*Streptomyces* sp. MM122	–	Cross-streak assays, VC assays	CIP, (24)
*Streptomyces* sp. MM128	–	Cross-streak assays	CIP, (24)
*Streptomyces coelicolor* M145	–	VC assays	JIC
*Streptomyces scabiei* 87-22	–	VC assays	JIC
*Streptosporangium roseum*	DSM 43021	VC assays	CIP
*Kocuria rhizophila*	ATCC 9341	VC assays	CIP
*Serratia marcescens*	ATCC 10759	VC assays	CIP
*Escherichia coli*	ATCC 25922	VC assays	CIP
*Citrobacter freundii*	ATCC 43864	VC assays	CIP
*Bacillus subtilis*	ATCC 19659	VC assays	CIP
Fungal and yeast strains			
*Aspergillus fumigatus*	Neqas 1210	Test strain in VC assays	CNR
*Candida albicans*	ATCC 10231	Test strain in VC assays	CNR
*Candida albicans ^R^*	13-160409-5014	Test strain in VC assays	CNR
*Candida glabrata*	ATCC 9030	Test strain in VC assays	CNR
*Candida krusei*	ATCC 6258	Test strain in VC assays	CNR
*Cryptococcus neoformans*	Neqas 2226	Test strain in VC assays	CNR
*Fusarium oxysporum*	13-180516-0034	Test strain in VC assays	CNR
*Fusarium solani*	Neqas 2650	Test strain in VC assays	CNR
*Penicillium chrysogenum*	Neqas 2068	Test strain in VC assays	CNR
*Rasamsonia argillacea*	Neqas 1872	Test strain in cross-streak and VC assays	CNR
*Lomentospora prolificans*	13-151125-0018	Test strain in VC assays	CNR

^*a*^ CIP, Center for Protein Engineering, University of Liège, B; JIC, John Innes Center, Norwich UK; CNR, Center National de Référence pour les Mycoses, University of Liège, B; *^R^*, azole-resistant.

^*b*^ “–” means that there is no culture collection number of these strains.

### Antimicrobial assays

#### Cross-streak assays for antifungal activity

Antimicrobial activity was assessed using cross-streak assays on solid media, following previously described methods (54). Each actinobacterial strain was independently inoculated from the mycelium stock as a single line in the center of the square Petri dish and incubated for 7 days at 28°C. A suspension of *R. argillacea* (OD₆₂₅ = 0.1 ± 0.02) was prepared in water from mycelium scraped from a 3-day culture (at 28°C on YM) and was inoculated with a cotton swab as a perpendicular streak against moonmilk isolates. The plates were incubated at 28°C, and the values of inhibition (in percentages), compared to the growth of the negative control, were recorded after 3 days.

### Volatile-compound-mediated growth-inhibition assay

The inhibitory effect of VCs on fungal growth was evaluated in two-compartment Petri dishes as previously described (55). One compartment was poured with the appropriate medium for bacterial growth, while the other contained YM agar for fungal growth. Bacteria were spread as a confluent lawn and incubated to allow VC accumulation. Dishes were sealed with two layers of Parafilm to minimize volatile loss, and temperature and time of incubation were adjusted depending on strains (7 days at 28°C for actinobacteria and 3 days at 37°C for other bacteria). Mycelium from 3-day-old YM precultures of each fungal strain was then resuspended in water and adjusted to OD₆₂₅ =0.10 ± 0.02. Serial dilutions (10², 10³, 10⁴, and 10⁶ CFU/mL) were prepared, and 5 µL of each dilution was spotted onto the YM-containing compartment. Following fungal inoculation, plates were incubated at 28°C for a further 72 h, after which fungal growth was recorded to quantify VC-mediated inhibition.

### Optimization of cultivation conditions for VC identification

To enable direct sample injection for GC × GC–TOFMS analysis without the need for compound extraction from solid media, the ability of VCs to be produced in and retained by the cultivation medium was evaluated when strains were grown in MHB, the liquid form of MHA (Fig. S1). *Streptomyces* sp. MM122 and *Amycolatopsis* sp. MMun171 were cultured in MHB, and the antifungal activity of their culture supernatants was tested using bipartite Petri dishes as described above. The culture supernatants from MHB-grown MM122 and MMun171 inhibited the growth of *R. argillacea*, whereas no inhibition was observed with supernatants from ISP2 liquid medium (Fig. S1), consistent with findings from solid MHA.

### Pure compounds activity assay

The antifungal activity of pure DMDS and DMTS compounds (Acros Organics) against *R. argillacea* was carried out in bipartite Petri dishes, using the same protocol as described for the VCs production assay, with small changes. Instead of bacteria on agar medium, a volume of 100 µL of the compound to be tested was added into the cap of a 2 mL tube placed in the center of the empty compartment. Dilutions from 10^0^ to 10^7^ of the pure compounds (DMDS or DMTS) were prepared by 1:10 serial dilutions in ethanol and tested. As controls, Petri dishes with the fungi exposed to empty caps or caps with ethanol were used. Each dish was immediately sealed with a double layer of Parafilm and placed in its own individual airtight container to completely eliminate the risk of cross-contamination. They were incubated for 3 days at 28°C, and fungal growth inhibition was checked after incubation.

### Comparative volatilomics

#### Quality control procedure

Prior to the analysis of VCs released by the bacteria, the repeatability and the stability of the analytical method were assessed. The QC procedure consisted of the injection of the Grob mixture in every batch of 20 samples in order to monitor analytical variations and correct bias if required. The Grob mixture (containing 2,3-butanediol [CAS#: 6982-25-8], decane [CAS#: 124-18-5], 1-octanol [CAS#: 111-87-5], undecane [CAS#: 1120-21-4], nonanal [CAS#: 124-19-6], 2,6-dimethylphenol [CAS#: 576-26-1], 2,6-dimethylaniline [CAS#: 87-62-7], methyl decanoate [CAS#: 110-42-9], methyl undecanoate [CAS#: 1731-86-8], methyl dodecanoate [CAS#: 111-82-0], and dicyclohexylamine [CAS#: 103-83-7]; Supelco, Bellefonte, PA, USA) injection covered the entire period of samples analysis.

In addition, a pooled QC sample was prepared by mixing 10 mL aliquots from each growth condition from each growth condition (including both bacterial cultures and blank media). From this pooled sample, 5 mL were injected with each batch. This QC procedure demonstrated the high stability of the GC × GC system over the duration of the experiment, confirming that observed VC profile variation was not due to analytical fluctuations, increasing the confidence in the obtained results. Consequently, minimal data pre-processing (e.g., autoscaling) was required prior to statistical analysis, preserving the integrity of the data.

### GC×GC-TOFMS method and sample analysis

The bacterial sample analysis was performed with Pegasus BT 4D GC×GC-TOFMS system with a thermal quad jet modulator (LECO Corp., St. Joseph, MI, USA) equipped with a CTC PAL RSI autosampler (CTC analytics, Switzerland). Volatile metabolites were extracted using a divinylbenzene/carboxen/polydimethylsiloxane fiber from Supelco (Bellefonte, PA, USA). The samples were incubated for 10 min at 40°C prior to fiber exposure, followed by analyte extraction for 30 min at the same temperature. The fiber was desorbed into the GC injector for 3 min at 250°C in split mode (1:10). For chromatographic analysis, the column set used consisted of a non-polar Rxi-5SilMS (30 m × 0.25 mm id × 0.25 µm d*_f_*) connected to a mid-polar Rxi-17SilMS (2 m × 0.25 mm id × 0.25 µm d*_f_*; Restek Corporation, Bellefonte, PA, USA). Helium was used as a carrier gas at a flow rate of 1.4 mL/min. The primary oven temperature program was 40°C (hold 1 min) ramped to 200°C at a rate of 5°C/min. A fast temperature ramp of 15°C/min to 270°C was applied to ensure system cleaning between successive analyses. The secondary oven temperature was set with an offset of +5°C above the primary oven, and the modulator offset was maintained at +20°C relative to the primary oven. A modulation period of 2.5 s was used. Mass spectra were acquired in the range of 40−500 *m/z* at a rate of 200 spectra/s. The ion source was maintained at 250°C. Data acquisition was performed using ChromaTOF software version 5.32 (LECO Corporation).

### Data pre-processing

Chromatograms were processed using ChromaTOF Tile to enable chromatogram comparison and significant feature identification. Briefly, each chromatogram was cut into retention time tiles, and the mass spectra within each tile were averaged. The resulting mass spectrum was then used for compound annotation and peak area integration. Semi-quantitative information extracted from each tile was used for downstream data processing. The resulting cleaned chromatograms were subsequently aligned, and the aligned peak table was exported and further processed using Excel, R Studio, and MetaboAnalyst platform.

### Compound identification and confidence levels

Compound identification was performed following the guidelines of the Metabolomics Standards Initiative, adapted for comprehensive two-dimensional gas chromatography (GC × GC) data. Four levels of identification confidence were defined, from level 1 (highest) to level 4 (lowest). Level 1 compounds were reliably identified through comparison with authentic commercial standards and were required to meet all of the following criteria: a first-dimension retention time deviation (ΔRT) of less than 20 s (in-house standard), a Reverse Similarity Index (RSI) greater than 800, and a retention index deviation (ΔRI) of less than 20. Level 2 included putatively annotated compounds identified by high spectral similarity to commercial libraries (MAINLIB, Replib, and NIST_RI), with RSI > 800 and ΔRI < 20, but without the use of a reference standard. Level 3 comprised compounds putatively assigned to a known chemical class based on spectral similarity (RSI > 700). Level 4 included compounds with tentative features observed in spectral data with low RSI, indicating low confidence in structural assignment.

## Data Availability

GC×GC-TOF-MS VCs data set presented in this study are openly available at https://zenodo.org/records/15918158 (DOI:10.5281/zenodo.15918158).

## SUPPLEMENTAL MATERIAL

Fig. S1 spectrum.02666-25-s0001.docx Optimization of cultivation conditions for VC identification.

ASM does not own the copyrights to Supplemental Material that may be linked to, or accessed through, an article. The authors have granted ASM a non-exclusive, world-wide license to publish the Supplemental Material files. Please contact the corresponding author directly for reuse.
