# Supplementary material for: Comparative volatilomics identifies ubiquitous sulfur compounds inhibiting the fungal pathogen Rasamsonia argillacea
Source: Microbiol Spectr. 2025 Nov 28;14(1):e02666-25. doi: 10.1128/spectrum.02666-25 (PMC12772349; doi:10.1128/spectrum.02666-25)
Supplement: Fig. S1 — Optimization of cultivation conditions for VC identification. [file spectrum.02666-25-s0001.docx]

**Supplemental material**


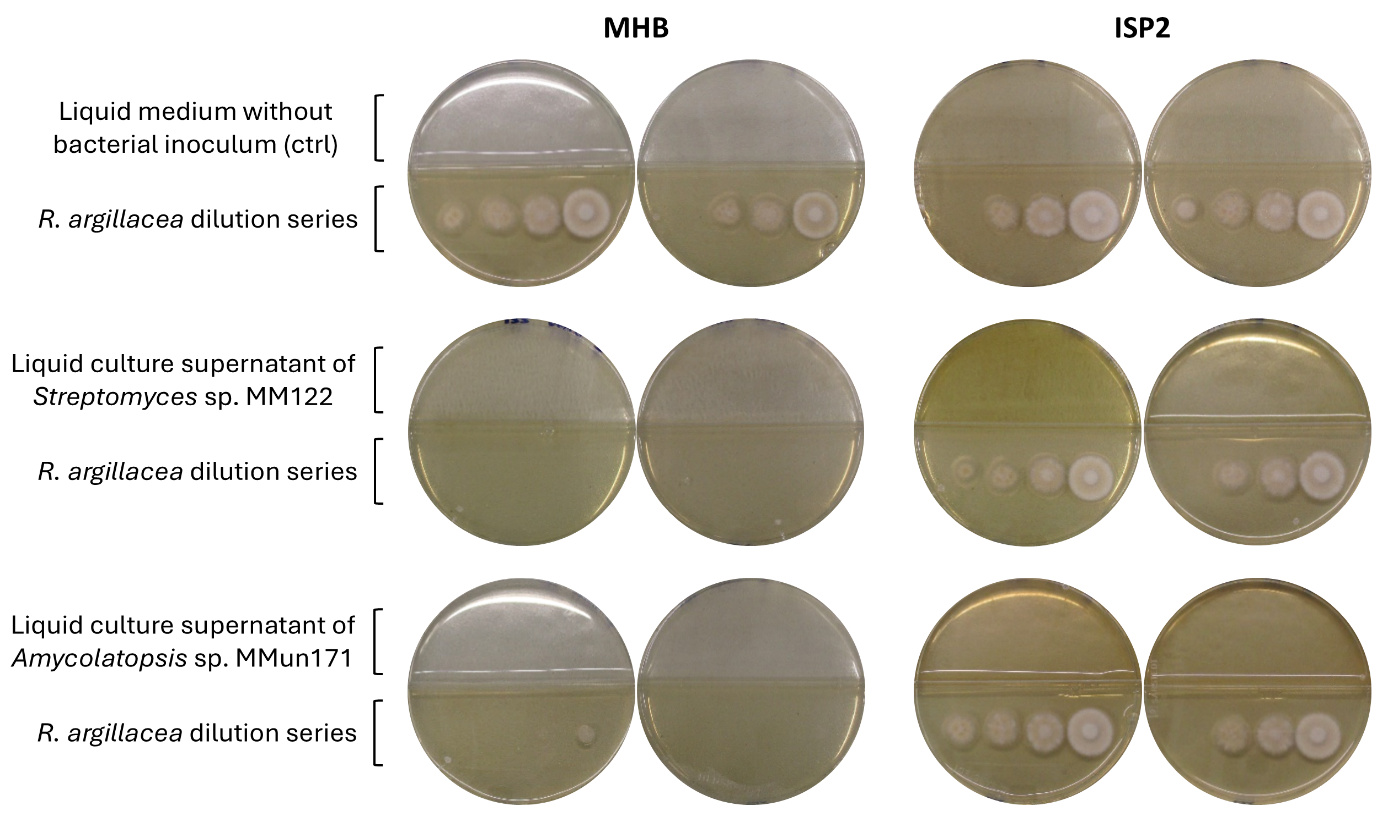
**Figure S1. Optimization of cultivation conditions for VC identification.** Evidence of production of volatile antifungal(s) active against *R. argillacea* in liquid MHA (MHB). The liquid culture supernatant of *Streptomyces* sp. MM122 and *Amycolatopsis* sp. MMun171 is poured in the top compartment of the bi-partite Petri dish while *R. argillacea* is inoculated in a four-spot dilution series (10^2^, 10^3^, 10^4^, and 10^6^ CFU/ml) in the other compartment on YM medium**.** MHB and ISP2 liquid media not inoculated with bacteria are used as controls (top line). The test was conducted in duplicate.
